# Limb Necrosis in a Lung Cancer Case Presenting with Widespread Thrombosis

**DOI:** 10.1155/2012/172952

**Published:** 2012-11-27

**Authors:** Eda Erdis, Oguz Karahan

**Affiliations:** ^1^Department of Radiation Oncology, Hatay Antakya State Hospital, 31040 Hatay, Turkey; ^2^Department of Cardiovascular Surgery, Medical School of Dicle University, 21280 Diyarbakir, Turkey

## Abstract

The malignancies are commonly associated with enhanced thrombotic vascular events. The thrombotic events are also increased in lung cancer subtypes. Even, the systemic mortal thrombotic disorders were reported in the literature. We report a case of a nonsmall-cell carcinoma patient who progressed with widespread thrombosis.

## 1. Introduction

Venous thromboembolic events are frequent in cancer patients. It was reported that venous thromboembolism is 4–6 times more frequent than the normal population in these patients. This problem, as well as reduced quality of life, important in itself, raises the risk of mortality [1, 2]. Cancer and its treatment can affect all steps of coagulation cascade: alteration in blood flow, damage of endothelial cells, and trigger of procoagulants [2].

According to current investigation of various forms of lung cancer of all histological types have been suggested to cause venous thromboembolism [3]. Additionally the incidence of venous thrombosis significantly increases during chemotherapy or radiotherapy and in the presence of distant metastases [3]. However widespread thromboembolic events are not common in these cases. This situation can affect the course of the treatment.

## 2. Case

**Figure 1:**
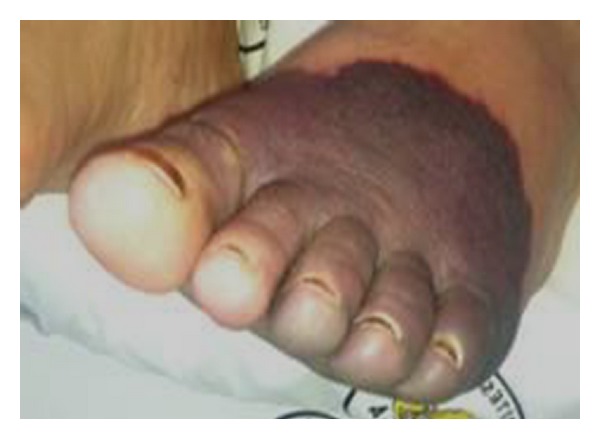
The cyanotic lesion in left foot.

A seventy-year-old female patient with an eight-year history of hypertension and a year of coronary artery disease was admitted to emergency department due to hands and feet edema. Additionally, she had a cyanosis in left foot (Figure 1). It was learned that her complaints increased since the last week. She explained additional progressive compressive chest pain with wheezing, cough, and palpitations in the last days. In physical examination, general condition was evaluated as moderate and bilaterally crackles in the basalis of the lungs; generalized limb and pretibial +2 edema was determined. All parameters detected normal levels except BUN and creatine in laboratory investigation. Higher urea and creatinine levels were evaluated in favor of acute renal failure. Afterwards, patient received hemodialysis program. Acute-subacute thrombosis was determined with color doppler ultrasound in right popliteal vein. Ejection fraction was evaluated as 30% and pulmonary artery pressure was evaluated as 60 mmHg in echocardiogram. Computed tomographic (CT) thorax angiography was performed for pulmonary thromboemboly suspicion. CT revealed bilateral minimal pleural effusion, consolide area, and a mass that extended into hilus and pleura in right lung upper lobe and lesion in left lung basal lobe posterobasal segment that were consistent with thrombus. Bronchoscopy was performed. In bronchoscopy, biopsy was performed from the right upper lobe entrance near the carinal differentiating where it was observed as mucosal tumoral infiltration. Upon arrival of pathological examination, results showed malign epithelial carcinoma, TTF-1 positive; pancytokeratin positive, it was reported as consistent with non-small-cell carcinoma, adenocarcinoma. Low molecular weight heparin (LMWH) therapy was started and chemotherapy was recommended. But the patient refused the treatment and she was discharged. After two weeks she was admitted to the emergency service with same complaints and additional bullous lesions and necrosis in her hands. Despite the antiaggregant and LMWH treatment, necrosis was observed in progress. Widespread venous thromboses were detected in control doppler ultrasound. She died in the second day of hospitalization, due to pulmonary edema.

## 3. Discussion

Patients with lung cancer are believed to have the highest risk of developing venous thrombosis during the course of their disease. But it was reported as adenocarcinoma is more often than other types for cause of thromboembolic events. Blom et al. studied 537 patients and they received similar results [3, 4]. In present case adenocarcinoma was detected according to pathology report.

Thrombosis is commonly seen in deep veins in lung cancer. However it can be identified in inferior and superior vena cava their main branches and may be identified in right cardiac compartments [5]. Additionally disseminate intravascular coagulation (DIC) may be developed [6]. Such a problem was detected in present case. Progressive thrombosis, necrosis, and bullous lesions were observed.

The main target of cancer treatment is removing the total disorder. But usually it is not possible. Because of this, treatment based primarily on increasing quality of life [5]. Preventing thromboembolic events which cause serious problems is important for improving the quality of life [7, 8]. Therefore the most appropriate thromboprophylaxis in cancer patients should be implemented [8]. LMWH treatment began in our case. But progressive thrombosis could not be blocked.

In conclusion thrombosis is an important problem in cancer patients. Varying intervals from deep vein thrombosis to DIC can occur. On the other hand these subsequent or accompanying problems influence response of treatment. For this precautions must be taken for thrombosis in cancer patients. We suggested that live quality can be upgraded if these preventable disorders can be clarified.
